# Use of Decision Support Tools to Empower Pregnant Women: Systematic Review

**DOI:** 10.2196/19436

**Published:** 2020-09-14

**Authors:** Elin Ngo, Maria Bich-Thuy Truong, Hedvig Nordeng

**Affiliations:** 1 PharmacoEpidemiology and Drug Safety Research Group Department of Pharmacy University of Oslo Oslo Norway; 2 Department of Child Health and Development Norwegian Institute of Public Health Oslo Norway

**Keywords:** decision support tools, pregnancy, mobile application, empowerment

## Abstract

**Background:**

Women face many health-related decisions during pregnancy. Digitalization, new technology, and a greater focus on empowering patients have driven the development of patient-centered decision support tools.

**Objective:**

This systematic review provides an overview of studies investigating the effect of patient-centered decision support tools for pregnant women.

**Methods:**

We searched 5 online databases, MEDLINE, EMBASE, Web of Science, PsycINFO, and Scopus, from inception to December 1, 2019. Two independent researchers screened titles, abstracts, and full-texts against the inclusion criteria. All studies investigating the effect of patient-centered decision support tools for health-related issues among pregnant women were included. Study characteristics and results were extracted using the review management tool Rayyan and analyzed according to topic, type of decision support tools, control group, outcome measurements, and results.

**Results:**

The 25 eligible studies covered a range of health topics, including prenatal screening (n=10), gestational diabetes and weight gain (n=7), lifestyle (n=3), blood pressure and preeclampsia (n=2), depression (n=1), asthma (n=1), and psychological well-being (n=1). In general, the use of decision support tools increased women's knowledge, and recording symptoms enhanced satisfaction with maternity care.

**Conclusions:**

The opportunities created by digitalization and technology should be used to develop innovative patient-centered decision support tools tailored to support pregnant women. Effect on clinical outcomes should be documented.

## Introduction

### Background

Patient-centered decision support tools are developed to involve patients in their own health-related decisions by (1) clearly stating the decisions that need to be made, (2) providing information about the options, outcomes, risks, and benefits, and (3) clarifying personal values. Decision support tools aim to complement, not replace, counseling from health care providers. The goal is to empower patients to make the decisions that are best for themselves and improve communication with their care providers [1,2].

Patient involvement in decision making varies among patient groups but is especially common among young women [3], coinciding with the time in life at which they become pregnant and, for many women, face completely new health-related decisions. In particular, decisions about medication use in pregnancy may be challenging, as it requires handling the unique task of weighing the benefits and risks of treatment for themselves against the benefits and risks for their unborn child. These situations are not uncommon, as over 60% of pregnant women use medications at least once during pregnancy [4-6].

Prior studies [7] have shown that pregnant women actively seek information to enable them to make decisions about medication use in pregnancy. First time pregnant women are more likely to seek information about medications and health-related problems during pregnancy than women who have previously had children [8-10]. Despite the frequent use of the internet, pregnant women tend not to discuss the information they have retrieved online with their health care providers [11]. Provision of tailored and credible information though a decision support tool may have the potential to empower and improve informed decision making among pregnant women [12].

The last literature review [13] on patient-centered tools to support women’s decisions during pregnancy was published in 2012. Since then, there has been an increased focus on digitalization and novel tools to empower patients. An updated literature review could help identify knowledge gaps concerning patient-centered decision support tools for pregnant women [14,15].

### Objective

The aim of this systematic review was to identify studies evaluating the efficacy of patient-centered decision support tools for pregnant women and provide guidance for future research and the development of new, efficient tools.

## Methods

### Literature Search Strategy

The following online databases were searched from inception to January 18, 2019: MEDLINE, EMBASE, Web of Science, PsycINFO, and Scopus. An updated search was conducted December 1, 2019. Each database was searched using a customized search strategy (Multimedia Appendix 1). The following keywords or MeSH terms (Medical Subject Headings) were used for the database search: *pregnancy*, *parturition*, *prenatal care*, *antenatal care*, *mobile application*, *mobile health*, *decision support techniques*, *choice behavior*, *patient education*, *decision making*, *satisfaction*, *quality of life*, and *knowledge*.

### Selection of Studies

The studies were selected in accordance with PRISMA (Preferred Reporting Items for Systematic Reviews and Meta-analyses) guidelines [16].

### Type of Study

Randomized controlled trials, cohort studies, register-based studies, and case-control studies were eligible for inclusion. Reviews, nonoriginal studies, Delphi studies, editorials, commentaries, letters to the editor, animal studies, and conference papers or abstracts were excluded. Full-texts in English were included in this review. Moreover, full texts in Norwegian, Swedish, or Danish were included, as the authors could fluently read papers in these languages.

### Type of Participants

All studies focusing on women who used one or several patient-centered decision support tools during pregnancy regarding health- or pregnancy-related issues were included in this review. Studies evaluating decision support tools for use in the prepregnancy period, postpartum period, or delivery-related (eg, support during birth, cesarean delivery, mode of birth after cesarean section, or breech position) were excluded.

### Type of Intervention

All types of tools (digital or paper-based) developed to support women’s health-related decisions by providing tailored information to her situation or recordings in pregnancy were included.

### Type of Control Group

Participants in the control group were pregnant women who received standard prenatal care or used a different decision support tool than the participants in the intervention group. A control group was not required in descriptive studies.

### Types of Outcome Measures

Outcome measures that assessed the women’s knowledge, satisfaction, decision making, quality of life, use experience, behaviors, or control of clinical measures in pregnancy were included.

### Study Selection and Data Extraction

All studies identified from the 5 databases were saved in reference management software (EndNote X8.1). Duplicates were removed, and the remaining studies were uploaded to free online systematic review data management software (Rayyan) [17]. First, the 2 researchers (EN and MT) independently screened titles and abstracts against the inclusion criteria, and disagreements were discussed until consensus was reached. The full-texts included from the previous round were then independently screened and categorized by the same researchers using EndNote and Excel (Microsoft Inc). At this step, excluded studies were categorized as (1) full-text not available, (2) foreign language, (3) wrong publication type, (4) wrong study design, (5) the study did not investigate the use of a decision support tool, or (6) the study did not include pregnant women or irrelevant outcome (eg, delivery, cesarean section, and economic analyses).

The studies included after the full-text screening were analyzed using a data extraction form (Multimedia Appendix 2). Information extracted from the studies included information about the study design, population, setting, method of recruitment, type of intervention or decision support tool, control group, outcomes measure, and results. Findings were grouped into major topics such as prenatal screening, gestational diabetes and weight gain, lifestyle, blood pressure and preeclampsia, depression, asthma, and physiological well-being.

## Results

### Search Findings

**Figure 1:**
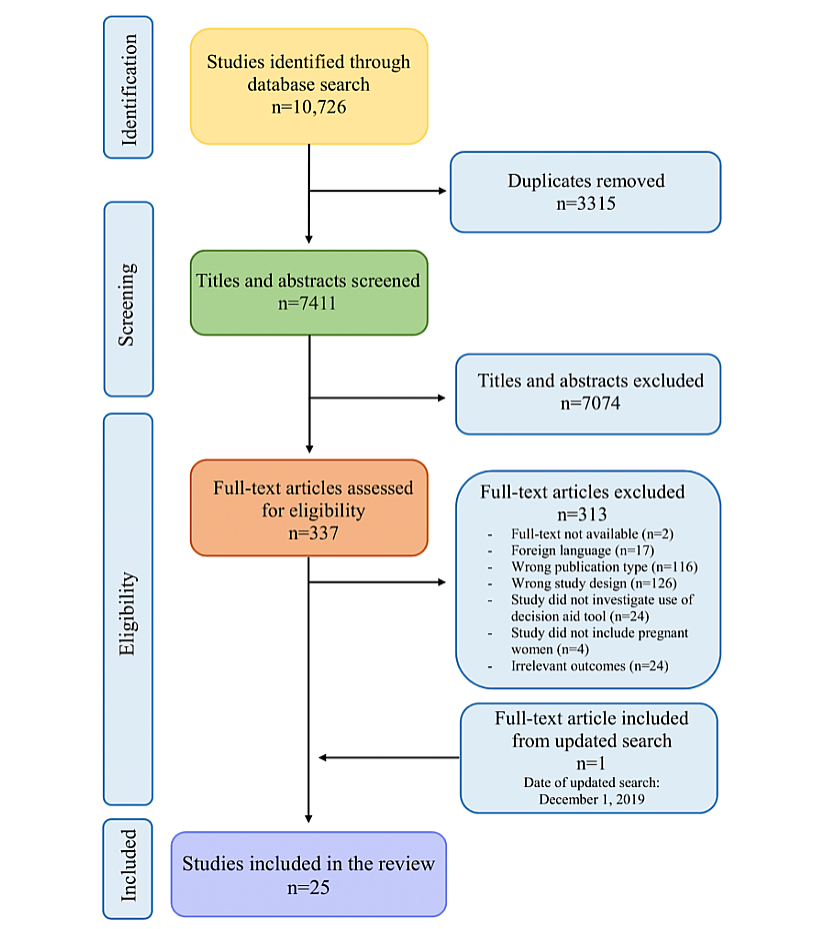
Flowchart of the identification and selection of evaluated studies.

A total of 10,726 studies were initially identified in the first search (January 18, 2019) from the 5 online databases, with 7411 remaining after the deletion of duplicates. Of these, 7074 studies were excluded based on titles and abstracts, and 337 full-texts were screened for eligibility (Figure 1). The most common reason for exclusion was wrong study design (n=126).

The updated search (December 1, 2019) identified 1221 new studies from the same databases as the first search. Of these, only 1 study was eligible for inclusion in this review after the screening process.

### Included Studies

**Figure 2:**
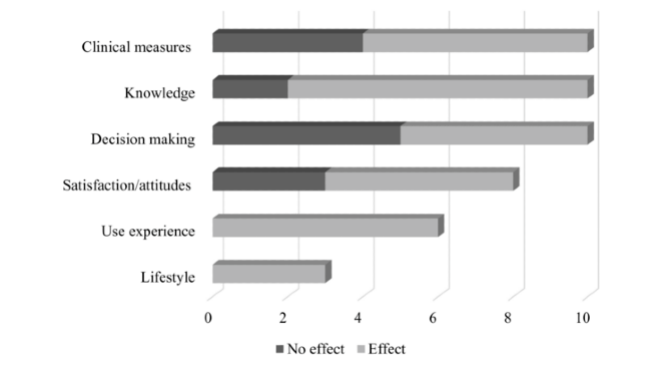
Effect of digital decision support tools.

A total of 25 studies were included in this review, all in English. The studies covered 7 major topics: prenatal screening, gestational diabetes and weight gain, blood pressure and preeclampsia, lifestyle, depression, asthma, and physiological well-being (Multimedia Appendix 3). The decision support tools were provided either as digital tools (webpage, mobile app, video, SMS text messages, n=24) or as written educational material (n=1). Outcome measures included in the digital decision support tools were clinical measures (n=10), knowledge level (n=10), decision making (n=10), satisfaction or attitudes (n=8), use experience (n=6), and lifestyle (n=3). One paper-based decision support tool investigated the effect on knowledge (n=1), attitudes (n=1), decision making (n=1), and clinical measures (n=2) (Figure 2). Several studies used multiple instruments for measuring the same outcome. The total number of outcome measures may thus exceed the number of studies included.

### Effect of Patient-Centered Decision Support as Interventions

#### Prenatal Screening

Ten studies [18-27] evaluated the effect of a patient-centered decision support tool on women’s decisions about performing prenatal screening for genetic disorders and birth defects. Pregnant women at ≤26 gestational weeks were included in these studies. One study [24] did not have a cut-off on gestational weeks. Nine decision support tools were digital and one was provided as written material. The outcomes measured in these studies were knowledge (n=9), decision making (n=11), satisfaction or attitudes (n=6), clinical measures (n=3), and use experience (n=1).

Overall, women who used a decision support tool had higher knowledge scores than the control group and knew about the risks and benefits of genetic screening in pregnancy (Multimedia Appendix 3). Independent of the type of decision support tool, the results show decreased decisional conflict for women in the intervention group compared to those in standard care. This indicated that women using decision support tools felt more informed and were more aware of the risk and expected outcome of each option when compared to their counterparts [19-21,23,27]. Women using decision support tools also had better knowledge scores [19,21-23,25-27], except for in 2 studies [20,24] which showed no effect on knowledge. Both digital and paper-based decision support tools showed no difference in attitudes and frequency of completing screening (digital: 32%; paper-based: 15%; *P=*.087) [19,23,25,27].

#### Gestational Diabetes and Weight Gain

Seven studies [28-33] investigated the effect of using decision support tools on blood glucose level control for pregnant women with gestational diabetes. Two studies [28,34] evaluated the effect on gestational weight gain in general and among women with gestational diabetes. The evaluated decision support tools were apps (n=4), web-based tools (n=2), and SMS text message–based (n=1). Outcome measures were knowledge level (n=1), satisfaction (n=2), use experience (n=2), blood glucose level control (n=3), and weight control (n=2).

Women using an app to record blood glucose level readings daily, in addition to receiving SMS text messages from their doctor with advice when readings were abnormal, reported more blood glucose level readings than women who recorded their blood glucose level readings in a paper diary (app: 3.8; paper diary: 2.6 recordings per day) [30]. The vast majority of women with diabetes using the apps felt more satisfied with the care they received [29]. Women receiving tailored advice online (about blood glucose) from their care provider also had a better understanding of the risks related to gestational weight gain for themselves (tailored advice: 34%; control: 21%; *P=*.044) and the fetus (tailored advice: 62%; control: 38%; *P=*.001) [31].

Women using apps as decision support tool showed no difference or improvements in in blood glucose level control [28,30]. However, women who used a web-chat with direct contact and feedback from their health care providers had significant lower fasting blood glucose level (web-chat and feedback: 4.3; control: 5.3; *P<*.001) and 2-hour postprandial blood glucose (web-chat and feedback: 5.8; control: 6.9; *P<*.001) [33]. They also felt they had more control of their symptoms and a better overview of their blood glucose when using a decision support tool as a supplement to standard care [32].

#### Lifestyle

Three studies [35-37] investigated the effect of decision support tools on alcohol consumption and smoking cessation during pregnancy. The tools were an app [35], a web-based tool [36], and an SMS text message–based tool [37].

A computer-tailored letter providing information about the risk of alcohol use in pregnancy had no effect on women’s refrainment from alcohol use after 3 months when compared to standard care. They did, however, refrain from alcohol to a larger extent after 6 months (computer-tailored letter: 78%; standard care: 55%, *P=*.04) [36]. Providing SMS text messages with general pregnancy information also resulted in a decreased alcohol consumption in pregnancy compared to maternity care alone (SMS text messages: 3.5%; standard maternity care: 1.1%; *P<*.098) [37].

#### Blood Pressure and Preeclampsia

Two studies [38,39] investigated the effect of an app on blood pressure readings and knowledge about preeclampsia. Women using the app recorded their blood pressure and shared the information with their care provider more frequently [38]. They also had significantly higher knowledge scores than women not using the app (app user: 78.1; control: 15.8; *P<*.001) [39].

#### Depression

A recently published study [40] investigated the effect of a mood tracking and alert app among pregnant women with depression on mood and depressive symptoms measured by the Patient Health Questionnaire 9 [41]. The app also provided information about mental health and physical activity and alerted prenatal providers when depressive symptoms were worsening. All women in the study also had access to a patient portal that provided an overview of upcoming appointments and clinical results and which could be used to request prescription refills. Women in the intervention group recorded depressive symptoms an average 5.3 days per week. Their health care providers were more likely to mention mental health at check-ups (*P=*.02), and women using the app had a higher rate of referral to a mental health specialist (*P=*.03) [40].

#### Asthma

One study [42] investigated the effect of an app on asthma symptoms during pregnancy. In that study, 58% of the women had moderate to severe asthma. Women in the intervention group received a chronic obstructive pulmonary disease measurement device (COPD‐6) in addition to an app for recording symptoms and medication use weekly, as well as with weekly feedback. Women in the intervention groups had better control of symptoms (Asthma Control Questionnaire: –0.30 vs. 0.06, *P=*.02), and quality of life (Asthma Quality-of-life Questionnaire score: 0.51 vs. –0.22, *P=*.002) after 6 months [42].

#### Psychological Well-Being

One study [43] investigated the use of a decision support tool and its effect on psychological well-being. Women received SMS text messages with information tailored to their gestational week, 2 times per week from gestational week 28 onward. Women receiving these SMS text messages had lower anxiety scores (2.8 vs. 4.9, *P=*.002) and higher confidence scores (8.9 vs. 7.8, *P=*.001) than women receiving standard care only [43].

## Discussion

### Main Findings

This systematic review provides an updated overview of current knowledge regarding patient-centered decision support tools for women during pregnancy. The 25 studies included more than 5000 women covering a broad range of health conditions in pregnancy. The majority of studies investigated the effect of a decision support tool in relation to prenatal screening (10/25, 40%) or gestational diabetes and weight gain during pregnancy (7/25, 28%). In general, the decision support tools were found to increase the women’s knowledge and enhance communication with health care providers. Digital decision support tools also seemed to be more convenient and led to more recorded clinical data than what was recorded by paper-based tools.

Interestingly, almost all decision support tools, both digital and written material, increased the women’s knowledge compared to knowledge received through standard care [19,21-27,31]. However, the majority of women participating in the studies were highly educated, and had been pregnant before; thus, they may not be representative of the general pregnant population. In addition, knowledge scores were most commonly measured immediately after the intervention was given or within 6 weeks. Therefore, whether gained knowledge lasted over time is unknown. One study [20] found no difference in knowledge between women receiving genetic counseling about prenatal screening with and without a supplementary app. The fact that both groups received a high-standard intervention such as genetic counseling could possibly explain why there was no additional benefit of the app on knowledge scores. Taken together, these results indicate that decision support during pregnancy, regardless of whether it is written or digital, may be a useful complement to standard antenatal care when specialized counseling is less available. It is still important to bear in mind that women receiving a consultation in advance may have been influenced to read more, which may have affected the results.

The studies included in this review show the potential of a patient-centered decision support tool to promote communication between health care providers and women. Women who frequently used digital support tools were more likely to bring their recordings to their health care provider. They were also more satisfied with the care they received and discussed their concerns with the health care provider to a greater extent than their counterparts did [27,29,31,38,40]. This indicates that women are more likely to discuss their problems with their health care providers when they are knowledgeable about the topic [44-46]. It should be noted that many of the studies included samples of women of higher sociodemographic status than that of the general population of pregnant women. This may have caused a selection bias of potentially more resourceful or motivated women, limiting the generalizability of the findings to all pregnant women.

### Interpretation in Light of Other Evidence

The use of decision support tools, in general, improves patient knowledge, make them better informed, and makes their choices and options clearer [47,48]. This review shows that this also applies to pregnant women. Mobile apps and decision support tools are increasingly used for self-management in many different chronic diseases that women of reproductive age have, such as migraine and diabetes, but high-quality decision support tools developed specifically for pregnancy are, to a large degree, still lacking. Moreover, there is clear potential for developing decision support tools to support decisions about medications in pregnancy. Nausea and vomiting in pregnancy, pain and self-managed conditions such as heartburn and constipation are examples where digital treatment algorithms may yet prove to be useful.

Our findings expand on and support earlier reviews that reported the potential benefits of decision support tools for decisions related to pregnancy. Both Say et al [49] and Dugas et al [13] advocated the potential for decision support tools to improve obstetric care. Our review included more studies that were recent (since 2012), even though our inclusion criteria were focused on decision support tools used only by women during pregnancy. More decision support tools after 2012 are electronic, as apps and web-based. The opportunities created by digitalization and technology should be used to develop innovative patient-centered decision support tools tailored to support pregnant women. Furthermore, the studies in our review covered a wider range of topics during pregnancy, but coverage of the most common topics regarding women’s health during pregnancy was still lacking (eg, decision support tools for nausea and vomiting in pregnancy).

### What Makes a Good Decision Support Tool for Pregnant Women?

The most effective decision support tools for pregnant women shared some common features. First, digital decision support tools seem more convenient if evidenced-based and if relevant information from different sources can be assembled in one app. This will avoid multiple or conflicting information sources, which has previously been an important concern among pregnancy women [50].

Second, digital tools that enable pregnant women to share recordings with their health care providers and get real-time feedback seem to be the most useful [18,29,32]. Such tools enable individually tailored information and improve communication during pregnancy. This is in line with previous findings on weight gain in pregnancy showing that specific and tailored information is more effective than general information [34].

Lastly, digital decision support tools were more convenient for recording symptoms than spiral notebooks. Women using digital support tools recorded their symptoms more frequently [38]. An earlier study [51] comparing the use of digital tools and spiral notebooks in general also reported that digital tools are potentially more accurate. This indicates that future development of decision support tools should focus and invest in digital tools.

### A Supplement, Not a Replacement

Even with increased technology, there is still a gap in the development of patient-centered decision support tools for pregnancy-related conditions. Given that women have high information needs and the potential that decision support tools have in empowering them, we expect this can be a valuable supplement for both women and their health care providers during prenatal care. Given that women were more satisfied with and were more likely to discuss their health problems with their care providers [30,31,38,40], it seems plausible that patient-centered decision support tools may promote healthier pregnancies and reduce the burden on health care services, with little extra cost after development. Decision support tools do not replace health care providers but provide additional relevant clinical information, supporting women to make better decisions together with their health care providers.

The sparseness of studies evaluating the effect of decision support tools, especially on clinical outcomes, stands in great contrast to the number of apps targeting pregnant women. This highlights the importance of developing and testing decision support tools for pregnant women. Only tools that are of high quality and that are efficient should be promoted.

### Limitations

This literature review has some limitations that should be taken into consideration when interpreting the results. First, there were few patient-centered decision support tools within each topic, and the diversity of outcome measures made it challenging to draw overall conclusions. Second, the individual studies overrepresented women with higher sociodemographic status, and the majority of pregnant women included in the studies were of a white ethnic background. Third, a number of studies had a low number of participants, and the women who consented to the studies may have been motivated to participate, which can cause a selection bias and give more positive results than what would be achieved in the typical target population.

Studies including decision support tools used by health care providers, decision support tools regarding childbirth, maternal and fetal health outcomes, and decision tools used in the postpartum period were excluded. An expanded review including these outcomes and topics should be assessed in future studies and may provide greater insight into the field.

### Conclusion

Despite the technological possibilities, the focus on patient involvement, and documented information needs, few heterogeneous studies have been performed on the effect of decision support tools in pregnancy. These few studies, however, have demonstrated the potential benefit to knowledge, perception, confidence in decision making, and communication between the women and their health care providers. More decision support tools should be developed and tailored to meet the needs of pregnant patients. The effect of such tools on clinical outcomes should be tested before recommending them or implementing them as a supplement in routine maternity care.

## Appendix

Multimedia Appendix 1 Search strategy.

Multimedia Appendix 2 Extraction sheet.

Multimedia Appendix 3 The characteristics of studies included in this review.
