# Supplementary material for: Use of Decision Support Tools to Empower Pregnant Women: Systematic Review
Source: J Med Internet Res. 2020 Sep 14;22(9):e19436. doi: 10.2196/19436 (PMC7522732; doi:10.2196/19436)
Supplement: Multimedia Appendix 1 [file jmir_v22i9e19436_app1.docx]

**Search strategy: MEDLINE**

| **Patient** | **Exposure** | **Outcome** |
| --- | --- | --- |
| **MeSH terms** | | |
| Pregnancy  Pregnant Women  Parturition  Prenatal Care | Decision Support Techniques  Mobile Applications  Smartphone  Decision Support Systems | Choice Behavior  Pregnancy Outcome  Patient Education as Topic  Decision Making  Personal Satisfaction  Patient Satisfaction  Quality Of Life  Patient Medical Knowledge  Patient Participation  Health Education  Clinical Decision Making |
| **All fields** | | |
| Pregnan*  Parturition  Childbirth  Birth*  “Prenatal care”  Antenatal care” | Mobile health  Decision support*  App*  Decision aid  Decision tool | Choice behavior  “pregnancy outcome”  Education  “decision making”  Satisfaction  “Quality of life”  Knowledge |

MeSH: Medical Subject Headings

Search January 18^th^ in PubMed/MEDLINE

**# search Results**

1 exp Pregnancy/ **851535**

2 exp Pregnant Women/ **7161**

3 exp Parturition/ **15285**

4 exp Prenatal Care/ **25129**

5 Pregnan*.mp. **951429**

6 Parturition.mp. **113007**

7 Childbirth.mp. **43816**

8 Birth*.mp. **352951**

9 “Prenatal care”.mp. **30293**

10 “Antenatal care”.mp. **7862**

11 1 OR 2 OR 3 OR 4 OR 5 OR 6 OR 7 OR 8 OR 9 OR 10 **1172761**

12 exp Decision Support Techniques/ **72548**

13 exp Mobile Applications/ **3674**

14 exp Smartphone/ **2518**

15 exp Decision Support Systems/ **7013**

16 Mobile health.mp. **51020**

17 Decision support*.mp. **33643**

18 App*.mp. **23489**

19 Decision aid.mp. **85529**

20 Decision tool.mp. **16996**

21 12 OR 13 OR 14 OR 15 OR 16 OR 17 OR 18 OR 19 OR 20 **182660**

22 exp Choice Behavior/ **51941**

23 exp Pregnancy Outcome/ **68580**

24 exp Patient Education as Topic/ **81298**

25 exp Decision Making/ **183398**

26 exp Personal Satisfaction/ **16575**

27 exp Patient Satisfaction/ **81975**

28 exp Quality Of Life/ **170736**

29 exp Patient Medical Knowledge/ **140**

30 exp Patient Participation/ **23327**

31 exp Heath Education/ **229616**

32 exp Clinical Decision Making/ **4737**

33 Choice Behavior.mp. **70120**

34 “Pregnancy putcome”.mp. **51778**

35 education.mp. **1360091**

36 “Decision Making”.mp. **185932**

37 Satisfaction.mp. **184173**

38 “Quality of life”.mp. **291509**

39 Knowledge.mp. **681905**

40 22 OR 23 OR 24 OR 25 OR 26 OR 27 OR 28 OR 29 OR 30 OR 31 OR 32
OR 33 OR 34 OR 35 OR 36 OR 37 OR 38 OR 39 **2557054**

41 11 AND 21 AND 40 **2225**

**Search strategy: EMBASE**

| **Patient** | **Exposure** | **Outcome** |
| --- | --- | --- |
| **Emtree** | | |
| Pregnancy  Pregnant Women  Birth  Prenatal Care  Childbirth | Decision Support System  Mobile Applications  Smartphone | Choice Behavior  Pregnancy Outcome  Patient Education as Topic  Decision Making  Personal Satisfaction  Patient Satisfaction  Quality Of Life  Patient Medical Knowledge  Patient Participation  Health Education  Clinical Decision Making |
| **Keywords** | | |
| Pregnan*  Parturition  “Prenatal care”  Childbirth  “Antenatal care” | Decision  Support  Techniques  Mobile  Application  Smartphone*  Decision  Support  System Health  Tool  Aid  App | Choice behavior  “pregnancy outcome”  Education  “decision making”  Satisfaction  “Quality of life”  Knowledge |

Search January 18^th^ in EMBASE

**# search Results**

1 exp Pregnancy/ **762703**

2 exp Pregnant Women/ **74071**

3 exp Birth / **28370**

4 exp Prenatal Care/ **145609**

5 exp Childbirth/ **60170**

6 Pregnan*.mp. **1026330**

7 Parturition.mp. **18182**

8 “Prenatal care”.mp. **40735**

9 Childbirth.mp. **33477**

10 “Antenatal care”.mp. **10304**

11 1 OR 2 OR 3 OR 4 OR 5 OR 6 OR 7 OR 8 OR 9 OR 10 **1118623**

12 exp Decision Support System/ **21973**

13 exp Mobile Applications/ **7446**

14 exp Smartphone/ **7329**

15 Decision.mp. **544262**

16 Support.mp. **1217546**

17 15 AND 16 **77691**

18 Tool.mp. **601376**

19 Aid.mp. **236666**

20 15 AND 18 **28490**

21 15 AND 19 **13481**

22 Techniques.mp. **2416332**

23 15 AND 16 AND 22 **6098**

24 Mobile.mp. **128926**

25 Application.mp. **918265**

26 24 AND 25 **17727**

27 Smartphone*.mp. **1896**

28 System*.mp. **6693570**

29 15 AND 16 AND 28 **41144**

30 Health.mp. **3731382**

31 24 AND 30 **25190**

32 App.mp. 29532

33 12 OR 13 OR 14 OR 17 OR 20 OR 21 OR 23 OR 26 OR 27 OR
29 OR 31 OR 33 **173207**

34 exp Medical Decision Making/ **82430**

35 exp Education/ **1397469**

36 exp Health Education/ **306258**

37 exp Pregnancy Outcome/ **52030**

38 exp Patient Education/ **106784**

39 exp Decision Making/ **340106**

40 exp Patient Satisfaction/ **125286**

41 exp Satisfaction/ **213759**

42 exp Knowledge/ **153535**

43 exp Quality Of Life/ **447634**

44 exp Patient Participation/ **24713**

45 Choice.mp. **393350**

46 Behavior.mp. **1403207**

47 45 AND 46 **29974**

48 “Pregnancy outcome”.mp. **63587**

49 “Decision making”.mp. **391759**

50 “Quality of life”.mp. **517707**

51 “Patient participation”.mp. **26195**

52 Education.mp. **1124943**

53 Knowledge.mp. **808589**

54 Satisfaction.mp. **256423**

55 34 OR 35 OR 36 OR 37 OR 38 OR 39 OR 40 OR 41 OR 42 OR 43 OR 44 OR
47 OR 48 OR 49 OR 50 OR 51 OR 52 OR 53 OR 54 **3247837**

56 11 AND 33 AND 55 **3918**

**Search strategy: PsycInfo**

| **Patient** | **Exposure** | **Outcome** |
| --- | --- | --- |
| **Thesaurus** | | |
| Pregnancy  Birth  Prenatal care | Decision Support System  Mobile Deceives | Satisfaction  Choice Behavior  Pregnancy Outcome  Client Education  Decision Making  Quality Of Life  Health Education  Health Knowledge |
| **Keywords** | | |
| Pregnan*  Parturition  Childbirth  “Prenatal care”  “Antenatal care” | Decision  Support*  Aid  Tool  App  “Smart phone*” | “Pregnancy outcome*”  Choice  Behavior  Education  Decision  Making  Satisfaction  “Quality of life”  Education  Knowledge |

Search January 18^th^ in PsycInfo

**# search Results**

1 exp Pregnancy/ **23515**

2 exp Birth/ **12786**

3 exp Prenatal Care/ **1933**

4 Pregnan.mp. **46933**

5 Parturition.mp. **1267**

6 Childbirth.mp. **5643**

7 “Prenatal care”.mp. **2970**

8 “Antenatal care”.mp. **840**

9 1 OR 2 OR 3 OR 4 OR 5 OR 6 OR 7 OR 8 **59547**

10 exp Decision Support System/ **3044**

11 exp Mobile Devices/ **6057**

12 Decision.mp. **157468**

13 Support*.mp. **450138**

14 Aid.mp. **36648**

15 Tool.mp. **86673**

16 12 AND 13 **32792**

17 12 AND 14 **2804**

18 12 AND 15 **5210**

19 App.mp. **5040**

20 “Smart phone*”.mp. **483**

21 10 OR 11 OR 16 OR 17 OR 18 OR 19 OR 20 **41464**

22 exp Satisfaction/ **56244**

23 exp Choice Behavior/ **26379**

24 exp Pregnancy outcome/ **16628**

25 exp Client Education/ **3734**

26 exp Decision Making/ **98126**

27 exp Quality Of Life/ **39218**

28 exp Health Education/ **17592**

29 exp Health Knowledge/ **7239**

30 “Pregnancy outcome*”.mp. **1958**

31 Choice.mp. **119474**

32 Behavior.mp. **863323**

33 31 AND 32 **41278**

34 Education.mp. **442108**

35 Making.mp. **249621**

36 12 AND 35 **114317**

37 Satisfaction.mp. **116647**

38 “Quality of life”.mp. **70535**

39 Education.mp. **442108**

40 Knowledge.mp. **290045**

41 22 OR 23 OR 24 OR 25 OR 26 OR 27 OR 28 OR 29 OR 30 OR 33 OR
34 OR 36 OR 37 OR 38 OR 39 OR 40 **957297**

42 9 AND 21 AND 41 **629**

**Search strategy: Scopus**

| **Patient** | **Exposure** | **Outcome** |
| --- | --- | --- |
| **All fileds** | | |
| Parturition  Pregnan*  Birth*  Childbirth*  Prenatal care  Antenatal care | “Decision support”  Mobile application*  Smartphone*  App  Decision aid  Decision tool | Choice behavior  “Pregnancy outcome*”  “Decision making”  Satisfaction  “Quality of life”  Knowledge  Patient participation  Education |

Search January 18^th^ in Scopus

**# search Results**

1 TITLE-ABS-KEY ( Parturition ) **26262**

2 TITLE-ABS-KEY ( Pregnan* ) **1072681**

3 TITLE-ABS-KEY ( Birth* ) **491181**

4 TITLE-ABS-KEY ( Child AND birth* ) **126747**

5 TITLE-ABS-KEY ( Prenatal AND care ) **63492**

6 TITLE-ABS-KEY ( Antenatal AND care ) **20146**

7 (TITLE-ABS-KEY ( Parturition ) OR (TITLE-ABS-KEY ( Pregnan* ) OR
(TITLE-ABS-KEY ( Birth* ) OR (TITLE-ABS-KEY ( Child AND birth* ) OR
(TITLE-ABS-KEY ( Prenatal AND care ) OR (TITLE-ABS-KEY ( Antenatal AND care) **1401082**

8 TITLE-ABS-KEY ( “Decision support” ) **107322**

9 TITLE-ABS-KEY ( Mobile application* ) **166573**

10 TITLE-ABS-KEY ( Smartphone* ) **47303**

11 TITLE-ABS-KEY ( app ) **42270**

12 TITLE-ABS-KEY ( Decision aid ) **29415**

13 TITLE-ABS-KEY ( Decision tool ) **133877**

14 (TITLE-ABS-KEY ( “Decision support” )) OR (TITLE-ABS-KEY ( Mobile application* )) OR (TITLE-ABS-KEY ( Smartphone* )) OR (TITLE-ABS-KEY ( app )) OR (TITLE-ABS-KEY ( Decision aid )) OR (TITLE-ABS-KEY ( Decision tool )) **466857**

15 TITLE-ABS-KEY ( Choice behavior ) **127044**

16 TITLE-ABS-KEY ( “Pregnancy outcome*” ) **69719**

17 TITLE-ABS-KEY ( “Decision making” ) **690386**

18 TITLE-ABS-KEY ( satisfaction ) **412006**

19 TITLE-ABS-KEY ( “Quality of life” ) **446306**

20 TITLE-ABS-KEY ( knowledge ) **1827128**

21 TITLE-ABS-KEY ( Patient participation ) **79066**

22 TITLE-ABS-KEY ( Education ) **185670**

23 (TITLE-ABS-KEY ( Choice behavior )) OR (TITLE-ABS-KEY ( “Pregnancy outcome*” )) OR (TITLE-ABS-KEY ( “Decision making” )) OR (TITLE-ABS-KEY ( satisfaction )) OR (TITLE-ABS-KEY ( “Quality of life” )) OR (TITLE-ABS-KEY ( Knowledge )) OR (TITLE-ABS-KEY ( Patient participation )) OR (TITLE-ABS-KEY ( Education )) **8452184**

24 ((TITLE-ABS-KEY ( Parturition ) OR (TITLE-ABS-KEY ( Pregnan* ) OR
(TITLE-ABS-KEY ( Birth* ) OR (TITLE-ABS-KEY ( Child AND birth* ) OR
(TITLE-ABS-KEY ( Prenatal AND care ) OR (TITLE-ABS-KEY ( Antenatal AND care)) AND ((TITLE-ABS-KEY ( “Decision support” )) OR (TITLE-ABS-KEY ( Mobile application* )) OR (TITLE-ABS-KEY ( Smartphone* )) OR (TITLE-ABS-KEY ( app )) OR (TITLE-ABS-KEY ( Decision aid )) OR (TITLE-ABS-KEY ( Decision tool ))) AND ((TITLE-ABS-KEY ( Choice behavior )) OR (TITLE-ABS-KEY ( “Pregnancy outcome*” )) OR (TITLE-ABS-KEY ( “Decision making” )) OR (TITLE-ABS-KEY ( satisfaction )) OR (TITLE-ABS-KEY ( “Quality of life” )) OR (TITLE-ABS-KEY ( Knowledge )) OR (TITLE-ABS-KEY ( Patient participation )) OR (TITLE-ABS-KEY ( Education ))) **2154**

25 ((TITLE-ABS-KEY ( Parturition ) OR (TITLE-ABS-KEY ( Pregnan* ) OR
(TITLE-ABS-KEY ( Birth* ) OR (TITLE-ABS-KEY ( Child AND birth* ) OR
(TITLE-ABS-KEY ( Prenatal AND care ) OR (TITLE-ABS-KEY ( Antenatal AND care)) AND ((TITLE-ABS-KEY ( “Decision support” )) OR (TITLE-ABS-KEY ( Mobile application* )) OR (TITLE-ABS-KEY ( Smartphone* )) OR (TITLE-ABS-KEY ( app )) OR (TITLE-ABS-KEY ( Decision aid )) OR (TITLE-ABS-KEY ( Decision tool ))) AND ((TITLE-ABS-KEY ( Choice behavior )) OR (TITLE-ABS-KEY ( “Pregnancy outcome*” )) OR (TITLE-ABS-KEY ( “Decision making” )) OR (TITLE-ABS-KEY ( satisfaction )) OR (TITLE-ABS-KEY ( “Quality of life” )) OR (TITLE-ABS-KEY ( Knowledge )) OR (TITLE-ABS-KEY ( Patient participation )) OR (TITLE-ABS-KEY ( Education ))) ANDDOCTYPE (le) AND DOCTYPE (cp) **1959**

**Search strategy for search in Web of Science**

| **Patient** | **Exposure** | **Outcome** |
| --- | --- | --- |
| **All fields** | | |
| Pregnan*  Parturition*  Prenatal care*  Childbirth*  Birth* | “Decision support*”  Mobile application*  Mobile health  App  Decision aid  Decision tool | Choice behavior  “Pregnancy outcome*”  “Decision making”  Satisfaction  “Quality of life”  Knowledge  Patient participation  Education |

Search January 18^th^ in Web of Science

**# search Results**

1 ALL FIELDS: ( Pregnan* ) **483361**

2 ALL FIELDS: ( Parturition* ) **18008**

3 ALL FIELDS: ( Prenatal care* ) **19892**

4 ALL FIELDS: ( Antenatal care* ) **13233**

5 ALL FIELDS: ( Childbirth* ) **99857**

6 ALL FIELDS: ( Birth* ) **394723**

7 #1 OR #2 OR #3 OR #4 OR #5 OR #6 **799153**

8 ALL FIELDS: ( “Decision support*” ) **60290**

9 ALL FIELDS: ( Mobile application* ) **116105**

10 ALL FIELDS: ( Smartphone* ) **10424**

11 ALL FIELDS: ( Mobile health ) **35204**

12 ALL FIELDS: ( App ) **66096**

13 ALL FIELDS: ( Decision aid ) **46968**

14 ALL FIELDS: (Decision tool) **103506**

15 #8 OR #9 OR #10 OR #11 OR #12 OR #13 OR #14 **391720**

16 ALL FIELDS: ( Choice behavior ) **94107**

17 ALL FIELDS: ( “Pregnancy outcome*” ) **25106**

18 ALL FIELDS: ( “Decision making” ) **324096**

19 ALL FIELDS: ( Satisfaction ) **203924**

20 ALL FIELDS: ( “Quality of life” ) **343338**

21 ALL FIELDS: ( Knowledge ) **1397807**

22 ALL FIELDS: ( Patient participation ) **46053**

23 ALL FIELDS: (Education ) **3205945**

24 #16 OR #17 OR #18 OR #19 OR #20 OR #21 OR #22 OR #23 **5153670**

25 #7 AND #15 AND #24 **1995**
