# Supplementary material for: Use of Decision Support Tools to Empower Pregnant Women: Systematic Review
Source: J Med Internet Res. 2020 Sep 14;22(9):e19436. doi: 10.2196/19436 (PMC7522732; doi:10.2196/19436)
Supplement: Multimedia Appendix 2 [file jmir_v22i9e19436_app2.docx]

**EXTRACTION SHEET -** Use of Decision Support Tools to Empower Pregnant Women: Systematic Review

**General information**

Reference number:

First author:

Year of data collection:

Year of publication:

Country:

Theme:

\
**Study design**


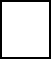

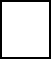

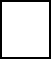

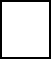
 Analytical study AND Randomized controlled study (RTC) Register-based study Other:_________________________________________


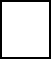

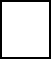

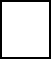
 Descriptive study Cohort study Case controlled study


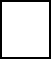
**Population**

Pregnant women Population size: _________________________________________ Other comments: __________________________________

**Setting**


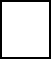

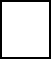

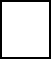

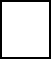
 Primary care Secondary care At home Other: _________________________________________

**Method of recruitment**


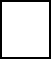

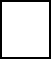

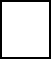

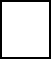
 By midwifes By physicians Internet/social media Other Other: _________________________________________

**Intervention**


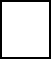

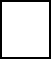

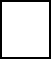

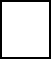
 Decision support tool as an app/on mobile Decision support tool on computer Decision support tool on paper

 Other: _________________________________________ Size of intervention group: _____________________________ Other comments: __________________________________


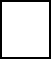
**Control**Type of control group: _______________________________ Size of control group: _________________________________ Other comments: __________________________________
 No control group

**Describe outcome:**

**Outcome**


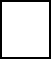

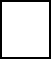

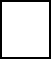

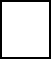

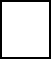

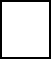
 Satisfaction Quality of life Knowledge Education


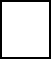

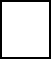
 Pregnancy outcome Choice behavior Decision making Other: _____________________________


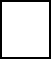

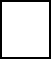
 **Included Excluded, reason:**________________________________________________
