# Supplementary material for: Use of Decision Support Tools to Empower Pregnant Women: Systematic Review
Source: J Med Internet Res. 2020 Sep 14;22(9):e19436. doi: 10.2196/19436 (PMC7522732; doi:10.2196/19436)
Supplement: Multimedia Appendix 3 [file jmir_v22i9e19436_app3.docx]

| Reference | Country | Study design | Population | DST intervention | Control | Outcome | Main results* |
| --- | --- | --- | --- | --- | --- | --- | --- |
| *Prenatal screening* | | | | | | | |
| Carlson et al., 2019 [20] | USA | RCT | Pregnant women, <22 weeks of gestation  I: n=92  C: n=105 | App as a DST and genetic counseling | Genetic counseling and standard care | Knowledge, 0-12 point scale (Higher score indicates higher level of knowledge) | No difference in knowledge (10.9 vs. 10.6, *P*=.306) between the groups |
|  |  |  |  |  |  | Decisional conflict, DCS, 10-item, 0-100 point scale (Lower score indicates less decisional conflict) | Decisional conflict was reduced among women using an app as a DST and women receiving genetic counseling (0.2 vs. 1.7, *P*=.003) |
| Rothwell et al., 2019 [25] | USA | RCT | Pregnant women, < 15 weeks of gestation  I: n=40  C: n=39 | Game-based decision tool | Brochure about prenatal screening, standard care | Knowledge, PSK survey, 23-item (Higher score indicates higher level of knowledge) | Higher knowledge score among women using the DST (21.4 vs. 19.6, *P*=.004) |
|  |  |  |  |  |  | Attitudes | More positive attitudes towards prenatal screening (no data reported) |
|  |  |  |  |  |  | Screening frequency (%) | Similar in frequency of completing screening between the groups (32% vs. 15%, *P*=.087). |
| Ahman et al., 2016 [18] | Sweden | Descrip-tive study | Pregnant women in second trimester after routine screening n=11 | Web-based decision tool | No control group | Women`s perception of a web-based decision tool | Enhanced awareness in prenatal screening. The information was more attractive, easier to access, and reliable cause it was selected. |
| Beulen et al., 2016 [19] | Nether-lands | RCT | Pregnant women with asthma, < 22 weeks of gestation  I: n=157  C: n=157 | Web-based decision tool | Standard care | Decisional conflict, DCS, 16-item, 0-100 point scale (Lower score indicates less decisional conflict) | Women using DST had lower decisional conflict (18.4 vs. 25.7, *P*=.002) |
|  |  |  |  |  |  | Decisional regret, DRS, 5-item, 0-100 point scale (Lower score indicates less decisional regret) | No difference in decisional regret between women using DST and standard care (14.0 vs. 14.5, *P*=.809) |
|  |  |  |  |  |  | Anxiety, STAI, 20-item, 0-80 point scale (Higher score indicates greater anxiety level) | No difference in anxiety between women using DST and standard care (10.2 vs. 10.3, *P*=.890) |
|  |  |  |  |  |  | Attitudes, MMIC, an attitude scale with five bipolar adjective pairs, scored as response towards having the procedures performed, midpoint of the scale equals neutral attitude  Scores above mid: positive attitude  Score below mid: negative attitude | No significant difference in attitudes between the two groups (15.6 vs. 16.5, *P*=.186) |
|  |  |  |  |  |  | Knowledge, 19 statements (true/false/do not know), scored as number of correct responses  ≥12: sufficient knowledge | Higher sufficient knowledge in the group using DST (14.9 vs. 12.8, *P*<.001) |
|  |  |  |  |  |  | Informed choice, MMIC, a combination of sufficient knowledge and choice value-consistent | Higher informed choice among women using DST (82.3% vs. 66.4%, *P*=.004) |
| Kuppermann et al., 2014 [22] | USA | RCT | Pregnant women, < 20 weeks of gestation  I: n=375  C: n=369 | Computerized decision support tool | Standard care | Knowledge, MSSKQ, 0-15 point scale (Higher score indicates higher level of knowledge) | Women using DST had higher knowledge score (9.4 vs. 8.6, *P*<.010) |
|  |  |  |  |  |  | Decisional conflict, DCS, 16-item, 0-100 point scale (Lower score indicates less decisional conflict) | No significant difference in decisional conflict between the groups (12.9 vs. 14.1, *P*=.470) |
|  |  |  |  |  |  | Decisional regret, DRS, 5-item, 0-100 point scale (Lower score indicates less decisional regret) | No difference on decisional regret between the groups (8.3 vs. 6.8, *P*=.120) |
| Skjoth et al., 2015 [24] | Denmark | RCT | Pregnant women  I: n=577  C: n=578 | Computer-based decision tool, videos, and chat forum | Standard care | Knowledge, MMIC, 10-item, 0-10 point scale  (Higher score indicates higher level of knowledge) | No difference in knowledge between the groups (8.3 vs. 8.2, *P*=.406) |
|  |  |  |  |  |  | Attitude, MMIC, 6-item survey, 0-36 point scale | No difference in attitude between the groups (33.7 vs. 33.5, *P*=0.433) |
|  |  |  |  |  |  | Informed choice, MMIC, measured by a combination of good knowledge, positive attitude and uptake of screening OR good knowledge, negative attitude, and no uptake of screening | No difference in making informed choice between the groups (91.8% vs. 93%, *P*=.588) |
| Yee et al., 2014 [26] | USA | RCT | Pregnant women, 6-26 weeks of gestation  I: n=75  C: n=75 | Computer-based education tool | Standard care | Knowledge, 23-item, 18 false/true and 3 open-ended questions, scored as the % of the items answered correctly | Women using DST had more correct answers (69.4% vs. 46%. *P*<.001) |
| Bjorklund et al., 2012 [27] | Sweden | RCT | Pregnant women, <11 weeks of gestation  I: n=236  C: n=247 | 25-minute film | Standard care | Knowledge, MMIC, 9-item, 0-9 point scale, scored as number of correct answers  5-9: sufficient knowledge | Women who watched the film had higher knowledge (6.9 vs. 6.4, *P*=.005 |
|  |  |  |  |  |  | Attitudes, MMIC, 6-item survey, 0-36 point scale | No difference in attitudes about screening between the groups (25.7 vs. 26.8, *P*=.275) |
|  |  |  |  |  |  | Informed choice, MMIC, a combination of good knowledge, positive attitude and uptake of screening OR good knowledge, negative attitude, and no uptake of screening | Women who watched the film made more informed choice about screening (71.5% vs. 62.4%, *P*=.062) |
| Kuppermann et al., 2009 [21] | USA | RCT | Pregnant women, <20 weeks of gestation  I: n=244  C: n=252 | Computerized decision tool | Education booklet | Knowledge, 10-item (Higher score indicates higher level of knowledge) | Significant higher knowledge among women using DST (77.6% vs. 65.5%, *P*<.001) |
|  |  |  |  |  |  | Satisfaction, 0-10 points scale | Women using DST had higher satisfaction (8.1 vs. 7.5, *P*>.001) |
|  |  |  |  |  |  | Decisional conflict, DCS, 16 questions, 0-100 points scale | Women using DST had lower decisional conflict compared to women receiving an education booklet (19.1 vs. 20.9, *P*=.21) |
| Nagle et al., 2008 [23] | Australia | Cluster RCT | Pregnant women, <12 weeks of gestation  I: n=218  C: n=221 | 24-page booklet | Standard care | Knowledge, MMIC, 8-item, 0-8 point scale  >4: Good knowledge  ≤4: Poor knowledge | More women receiving an education booklet had “good” level of knowledge compared to women following standard care (88% vs. 72%) |
|  |  |  |  |  |  | Informed choice, MMIC, measured by a combination of good knowledge, positive attitude and uptake of screening OR good knowledge, negative attitude, and no uptake of screening | More women receiving an education booklet made an informed choice (76% vs. 65%) |
|  |  |  |  |  |  | Decisional conflict, DCS, 16-item score, 0-100 point scale (Lower score indicates less decisional conflict) | No differences in level of decisional conflict. Mean score was low in both groups (1.7 vs. 1.7) |
|  |  |  |  |  |  | Attitudes, MMIC, 5-25 points score | No differences in attitude towards prenatal testing between the groups (86% vs. 81% had positive attitudes) |
|  |  |  |  |  |  | Anxiety, STAI, 6-item, 0-80 point scale (Higher score indicates greater anxiety level) | No differences in anxiety between the groups (36.2 vs. 37.4) |
|  |  |  |  |  |  | Depression, EPDS, 0-30 point score  ≥13: clinically depressed | No differences in depression between the groups (≥13: 11.6% vs. 11.2%) |
| *Gestational diabetes and weight gain* | | | | | | | |
| Guo et al., 2018 [28] | China | RCT | Pregnant women with gestational diabetes , 24-28 weeks of gestation  I: n=64  C: n=60 | App to record BG, provide information and notice when BG record was abnormal | Conventional outpatient treatment regimen | BG, FBG and PBG (mmol/L) | No significant improvement in BG among women using DST (*FBG*: 4.2 vs. 4.3,*P*=.602, *PBG*: 7.0 vs. 7.1, *P*=.683) |
|  |  |  |  |  |  | Weight gain (kg) | Less weight gain in the group using DST (3.2kg vs. 4.8kg, *P*<.001) |
| Mackillop et al., 2018 [30] | UK | Single center RCT | Pregnant women diagnosed with gestational diabetes, < 35 weeks of gestation  I: n=103  C: n=103 | App to record, tag, and review BG readings, and SMS (Short Message Service) with advice and encouragement | Standard care with a paper diary to record BG | Rate of BG change (mmol/L) and HbA_1c_ rise (%) | No significant difference in rate of BG change (-0.16 vs. -0.14, *P*=.78) and HbA_1c_ rise (0.02% vs. 0.03%) |
|  |  |  |  |  |  | Satisfaction, OMDTSQ, 9-item, 0-54 point scale  (Higher score indicates higher satisfaction) | Women from both group reported high satisfaction with the care they received (43.0 vs. 44.5, *P*=.049) |
| Skar et al., 2018 [32] | Norway | Descriptive | Women in the postpartum period who used the *Pregnancy+* app during pregnancy  n=17 | App to record, tag, and review BG readings | No control group | Experience | Easily accessible and trustworthy information, overview of BG increased feeling of control of symptoms, conflicting feedback between the app and HCP |
|  |  |  |  |  |  | Self-management | The app may have potential for assisting women in self-managing BG. |
| Yang et al., 2018 [33] | China | RCT | Pregnant women with gestational diabetes  I: n=57  C: n=50 | We-chat platform | Standard care | BG, FBG and PBG (mmol/L) | Significantly lower FBG and PBG among women using We-chat (*FBG*: 4.3 vs.5.3,*P*<.001, *PBG*: 5.8 vs. 6.9, *P*<.001) |
| McDonald et al., 2015 [31] | Canada | Cohort study | Pregnant women, < 20 weeks of gestation  I: n=131  C: n=310 | Web-based decision tool | Standard care | Knowledge of risk assessments | Women using DST discussed GWG-related topics with their health care provider more often (60.5% vs. 29.2%, *P*<.001). They also had higher knowledge about risk assessments:  *Risk in gaining excess GWG to themselves*: 79% vs. 50%, *P*=.014  *Risk in gaining excess GWG to their infants:* 64% vs. 56%, *P*=.295  *Risk in gaining inadequate GWG to themselves:* 34% vs. 21%, *P*=.044  *Risk in gaining inadequate GWG to their infants:* 62% vs. 38%, *P*=.001 |
| Hirst et al., 2014 [29] | UK | Descrip-tive study | Pregnant women with gestational diabetes, < 34 weeks of gestation | App to record BG and receive calls from doctor | Standard paper-based recording of BG | Satisfaction, OMDTSQ, 9-item  -3: strongly disagree  +3: strongly agree | The majority of women agreed or strongly agreed that the app was convenient and reliable |
| Pollak et at., 2014 [34] | USA | RCT | Overweight or obese pregnant women  I: n=23  C: n=12 | SMS 3 times/week with advice/ encouragements. Women received feedback based on their loggings | SMS three times/  week with general pregnancy information | Weight gain (kg) | Mean weight gain was 2.7kg less in the group receiving SMS with advice/encouragements |
| *Lifestyle* | | | | | | | |
| Dotson et al., 2017 [35] | USA | Descrip-tive | Pregnant women  n=210 | App with health education | No control group | Comprehension | Apps and other electric health education methods are useful and have potential for promoting tobacco cessation efforts in clinical setting. |
| van der Wulp et al., 2014 [36] | Nether-lands | Cluster 3-arm RCT | Pregnant women  I1: n=116  I2: n=135  C: n= 142 | I1: Computer-tailored letter of feedback after usual counseling from midwife  I2: Health counseling from midwife according to a given health counseling protocol | Standard care | Refrained from alcohol use, QFV-questionnaire | 3 months follow up: 65% of health counseling responders (vs. standard care, *P*=.790), 70% computer-tailored responders (vs. standard care, *P*=.150), and 45.4% standard care responders (vs. computer-tailored, *P*=.230) refrained from alcohol. These results were not significant.  6 months follow up: computer-tailored feedback can be effective to stop alcohol use during pregnancy, compared to standard care (78% vs. 55%, *P*=.04). Health counseling did not have an effect compared to standard care (72% vs. 55%, *P*=.26). |
| Evans et al., 2012 [37] | USA | RCT and descriptive | Pregnant women  n=123 | SMS with pregnancy information and tips | Standard care | Smoking | Women who reported smoking in the last 30 days decreased from 5.8% to 1.2% |
|  |  |  |  |  |  | Alcohol use | Women who reported consuming alcohol after they found out they were pregnant decreased from 3.5% to 1.1% (*P*<0.098) |
| *Blood pressure and preeclampsia* | | | | | | | |
| Ledford et al., 2017 [38] | USA | RCT | Pregnant women  I: n=120  C: n=121 | App with education material and recordings of BP | Spiral book with education material and BP recordings | Use of app | Women using the app recorded BP and shared the recordings with their health care providers more frequently (*P*<.001) |
| Parsa et al., 2019 [39] | Iran | Two groups | Pregnant women I: n=54  C: n=54 | App with information and education material | Standard care | Knowledge, 32-item (Higher score indicates higher level of knowledge) | Significantly higher knowledge score among women after use of DST (78.1 vs. 15.8, *P*<.001) |
| *Depression* | | | | | | | |
| Hantsoo et al., 2018 [41] | USA | RCT | Pregnant women with depressive symptoms, <32 weeks of gestation  I: n=48  C: n=24 | Mood tracking and alert (MTA) mobile app + patient “portal” app | Patient portal app | Patient engagement and care satisfaction, 6-item, Likert-scale from “very poor” to “excellent” OR “Completely agree” to completely disagree” | 41% of women in the MTA groups received a phone call from their providers, and had a higher rate of referral to a mental health specialist (*P*=.03). Their providers were also more likely to mention mental health (*P*=.02). No difference in confidence of managing their own health between the groups (6.1 vs. 6.1, *P*=.87) |
| *Asthma* | | | | | | | |
| Zairina et al., 2016 [42] | Australia | Pro-spective multi-center single-blinded RCT | Pregnant women with asthma and < 20 weeks of gestation  I: n=36  C: n=36 | COPD-6 to measure lung function. App to record asthma symptoms and give automated feedback message | Standard care | Change of symptoms after 3 and 6 months ACQ, 7-item, 0-6 point scale  0: totally controlled  6: severe uncontrolled | Women using DST did not have better control of symptoms 3 months after baseline (-0.01 vs. 0.16, *P*=.260), however they did have better control of symptoms 6 months after baseline (-0.30 vs. 0.06, *P*=.020) |
|  |  |  |  |  |  | Change in lung function after 3 and 6 months, measured by FEV_1_/FEV_6_ | No difference between the groups after 3 and 6 months (*3 months*: 3.43 vs. 0.14, *P*=.05, *6 months*: 1.53 vs. -0.56, *P*=.16) |
|  |  |  |  |  |  | Change in quality of life after 3 and 6 months, mAQLQ, 15-item, 0-7 point scale (Higher score indicates better quality of life) | No difference between the groups after 3 months (0.09 vs. -0-17, *P*=.15). The intervention group had a higher change in QOL after 6 months (0.51 vs. -0.22, *P*=.002) |
| *Psychological well-being* | | | | | | | |
| Jareethum et al., 2008 [43] | Thailand | RCT | Pregnant women < 28 weeks of gestation  I: n=32  C: n=20 | SMS 2 times/week and a phone call at gestational week 32 | Standard care | Satisfaction, questionnaire, 1-10 points scale (Higher score indicates higher satisfaction) | Women receiving SMS had a higher satisfaction score than women following standard care (9.3 vs. 8, *P*<.001) |
|  |  |  |  |  |  | Confidence, questionnaire, 1-10 points scale (Higher score indicates more confidence) | The group receiving SMS 2 times a week was significant more confidence (8.9 vs. 7.8, *P*=.001) |
|  |  |  |  |  |  | Anxiety, questionnaire, 1-10 points scale (Higher score indicates greater anxiety) | Women receiving SMS had lower anxiety score than women who did not receive SMS (2.8 vs. 4.9, *P*=.002) |

**RCT**=randomized controlled trial, **I**=intervention group, **C**=control group, **vs.**=versus, **DST**=decision support tool, **App**=mobile application, **p**=p-value, **DCS**=decision conflict scale, **PSK**=prenatal screening knowledge, **DRS**=decision regret scale, **STAI**=Speilberger State Trait Anxiety Inventory, **MMIC**=multidimensional measure of informed choice, **MSSKQ**=Maternal Serum Screening Knowledge Questionnaire, **EPDS**=Edinburgh Postnatal Depression Scale, **GWG**=gestational weight gain, **BG**=blood glucose, **FBG**=Fasting blood glucose, **PBG**=2-hour postprandial blood glucose, **OMDTSQ**=Oxford Maternity Diabetes Treatment Satisfaction Questionnaire, **SMS=**Short Message Service, **HCP**=Health care personnel, **HEI**=Healthy Eating Index, **QFV**=Dutch Quantity-Frequency-Variability, **BP**=blood pressure, **PP**=patient portal, **CORD**=chronic obstructive pulmonary disease measurement device, **FEV1/FEV6**=Forced expiratory volume in 1/6 s, **ACQ-7**=7-item Asthma Control Questionnaire, **mAQLQ**=Juniper`s mini-Asthma Quality-of-life Questionnaire score

**All main results is presented as mean scores.*
